# Lesion mapping in neuropsychological research: A practical and conceptual guide

**DOI:** 10.1016/j.cortex.2023.10.001

**Published:** 2023-10-20

**Authors:** Margaret J. Moore, Nele Demeyere, Chris Rorden, Jason B. Mattingley

**Affiliations:** a Queensland Brain Institute, The University of Queensland, St. Lucia, Australia; b Nuffield Department of Clinical Neurosciences, University of Oxford, Oxford, United Kingdom; c Department of Psychology, University of South Carolina, Colombia, SC, USA; d School of Psychology, The University of Queensland, St. Lucia, Australia

**Keywords:** Brain imaging, Lesion mapping, Braine–behaviour relationships, Neuropsychology, Lesion analysis

Lesion mapping is a popular method in neuropsychology in which the location of brain lesions in an individual or group of patients is used to infer braine–behaviour relationships (Bates et al., 2003). Broadly, statistical lesion mapping approaches aim to identify brain areas (e.g., voxels, regions of interest (ROIs), or tracts) which, when damaged, are associated with a specific behavioural impairment. Lesion mapping has advantages over common correlational imaging analyses (e.g., functional magnetic resonance imaging (fMRI), volumetric analyses), as lesion mapping can identify brain regions that are necessary for (not just involved in) regulating specific cognitive functions (Rorden & Karnath, 2004). Lesion mapping has been widely used across a range of disciplines and has played a fundamental role in establishing understanding of functional specialisation within the brain. Lesion mapping methods have evolved rapidly, expanding from early voxel-level univariate approaches toward more computationally intensive multivariate and network-level approaches. While these new methods offer novel insights into braine–behaviour relationships, lesion mapping can only yield informative conclusions if one asks the right questions and if the methods are applied correctly. Similarly, the generalisability of lesion mapping results ultimately depends on the quality of the analysis design, which can vary dramatically.

In this Viewpoint article, we provide an overview of the recent history and development of lesion mapping methods, with a special focus on work published in *Cortex*. We then provide a conceptual overview of important theoretical considerations for lesion mapping analyses, identify analysis parameters that affect analysis reliability, and highlight promising clinical applications. We then synthesise these topics and provide guidelines for designing high-quality lesion mapping studies. Finally, we highlight some key unanswered questions and important future directions. Our overarching goal is to provide clear and practical guidance for those wishing to conduct lesion mapping investigations, and to promote the design of high-quality and theoretically meaningful investigations into the relationships between brain and behaviour.

## 1. A short history of lesion symptom mapping in neuropsychology

**Fig. 1 –:**
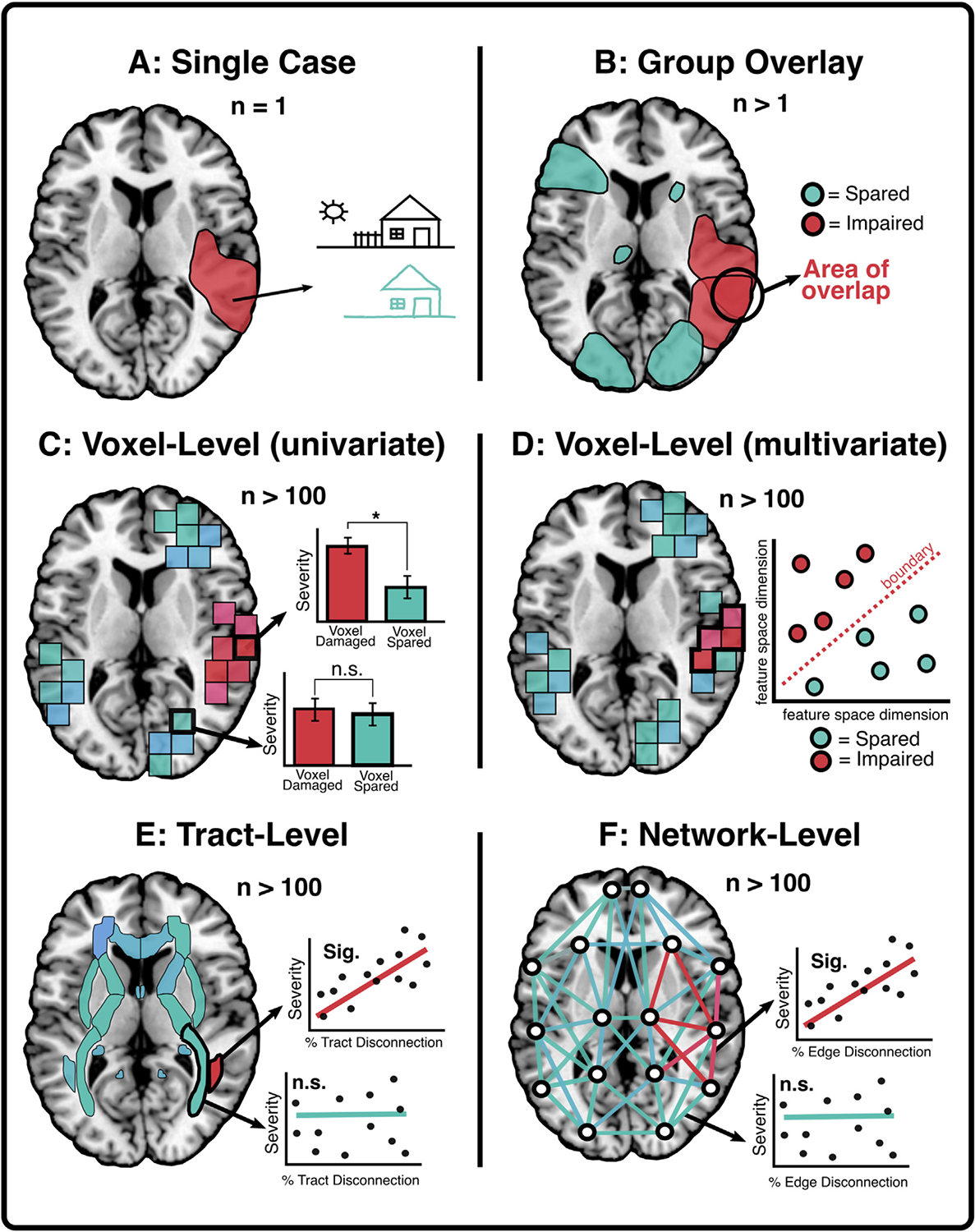
Overview of common lesion-mapping methodologies. These examples provide illustrations of different statistical approaches applied to investigate the neural correlates of visuospatial neglect. (A) Single case approaches infer that, in patients exhibiting a specific deficit (e.g., visuospatial neglect), the lesioned area (schematised in red), must be necessary for supporting the impaired function. (B) Group overlay approaches aim to identify areas of lesion overlap in groups of patients with a deficit of interest. Lesioned areas which are common across groups of impaired patients are assumed to represent critical neural correlates. (C) Univariate voxel-level approaches use mass-univariate statistical tests to identify voxels that, when damaged, are significantly associated with impairment. (D) Multivariate voxel-level methods quantify the synergistic contributions of multiple voxels (features) to build a best-fit impairment prediction model and identify regions which significantly contribute to this prediction. (E) Tract-level lesion mapping identifies white matter pathways for which the level of lesion-induced disconnection severity is significantly associated with the deficit of interest. (F) Network-level lesion mapping identifies brain network-level connections for which the level of lesion-induced disconnection is significantly associated with impairment severity.

Lesion mapping encompasses a broad range of methods where the goal is to use delineated areas of damage to elucidate braine–behaviour relationships (Fig. 1). Modern lesion mapping approaches evolved from classical single-case studies of patients with reliable patterns of behavioural impairment following damage to specific brain areas. Paul Broca’s (1861) report on patient “Tan” is a seminal example of the single-case lesion approach, linking aphasia with damage to left hemisphere fronto-temporal regions. Other classical single case studies include Wernicke’s (1874) report on fluent aphasia, Hughlings-Jackson’s (1888) pioneering work on epilepsy, Poppelreuter’s (1917) studies of visual impairments in soldiers injured during World War 1, and Luria’s (1947) work on aphasia in traumatic brain injury. Similar single-case approaches are still commonly used to help provide preliminary insight into the neural underpinnings of rare or unique neuropsychological syndromes (e.g., Moore & Demeyere, 2020 (Cortex); Ranzini et al., 2023 (Cortex)).

With the advent of non-invasive brain imaging methods like X-ray and CT scanning, group-level lesion comparison studies emerged, where the aim was to identify the most commonly damaged brain areas associated with specific behavioural impairments in groups of patients. For example, Warrington and Taylor (1973, Cortex) conducted a basic lesion comparison analysis to determine whether damage to right or left hemisphere anterior, temporal, or posterior areas was significantly associated with object recognition abilities. Specifically, the authors used t-tests to compare object recognition scores between patients with similar lesion locations across hemispheres and concluded that perceptual classification is impaired after lesions to right posterior cortical areas. Such approaches are still commonly used. For example, Barbey et al. (2013, Cortex) conducted a lesion location comparison to determine whether the dorsolateral prefrontal cortex (dlPFC) is necessary for working memory. The authors grouped 199 combat veterans according to whether or not their lesions overlapped with the dlPFC (as defined in a normative atlas) and conducted analysis of variance (ANOVA) to determine whether dlPFC, non-dlPFC, and control groups differed in terms of performance on memory tasks. They found that dlPFC damage was associated with deficits in maintaining and manipulating information in working memory (Barbey et al., 2013, Cortex). While lesion-comparison approaches can provide important preliminary insights into the involvement of particular brain areas in behaviour, their reliance on broad location categorisations can mask critical between-patient variability in lesion location. For example, lesion overlay approaches are inherently descriptive and cannot make claims about whether a given area is statistically related to a deficit. Likewise, lesion overlap analyses do not control for effects of lesion size, meaning that these analyses are inherently biased toward identifying areas impacted by larger lesions (DeMarco & Turkeltaub, 2018).

To address this issue, statistical voxel-based lesion mapping was developed. In a pioneering study, Bates et al. (2003) provided a novel demonstration of how mass univariate, voxel-level statistics could be applied to quantitatively identify specific voxels which, when damaged, are associated with behavioural impairments. This approach conferred an important methodological advancement by identifying statistical relationships between lesion location and behaviour. Critically, Bates et al.’s (2003) proposed method offered the option to statistically consider covariates in lesion analyses (e.g., lesion volume, age), thereby addressing a key, potentially confounding limitation present in group overlay lesion analyses. In the approach introduced by Bates et al. (2003), patients are categorised according to whether their lesion overlaps with each voxel. For each of these voxel-wise groupings, statistical tests (usually t-tests or Liebermeister analyses) are conducted to identify voxels for which there is a significant difference in the behavioural scores between patients with and without damage to that voxel. Following statistical adjustments for multiple comparisons and lesion volume, this method yields voxel masks that identify regions which, when damaged, are associated with the deficit of interest.

This mass univariate approach is currently the most popular lesion mapping technique and has been used to explore the neural correlates of a wide range of cognitive functions. It has been used to identify correlates of single deficits, to distinguish between regions associated with multiple, potentially dissociable impairments, and to quantify lesion patterns associated with poor recovery. Many of the seminal studies employing univariate lesion-mapping approaches have appeared in *Cortex*. For example, Varjačićet al. (2018) employed mass univariate lesion mapping to identify the neural correlates of executive set-switching and found that poor performance on a trail-making task was associated with damage to the left insula. Chechlacz et al. (2013) differentiated areas associated with visual and tactile extinction, visual neglect, and visual extinction. Dressing et al. (2020) employed mass univariate lesion mapping to identify the correlates of apraxia following left hemisphere stroke, and to identify lesion patterns associated with poor recovery from apraxia. Suarez et al. (2020) found that damage to dorsal stream areas of the right hemisphere was associated with viewer-centred neglect. In a study of individuals with left hemisphere injury, Stark et al. (2019) found semantically related paraphasias were associated with damage to anterior regions, whereas unrelated paraphasias involved more posterior injuries. Fridriksson et al. (2015) demonstrated that injury to the left insula correlated with spontaneous speech scores, whereas injury to the left inferior frontal region helped distinguish between patients who did and did not respond well to speech entrainment therapy. The examples above are merely a subset of the many applications of mass univariate lesion mapping in neuropsychology and are enumerated here to highlight the important role *Cortex* has played in promulgating this important field of research.

**Fig. 2 –:**
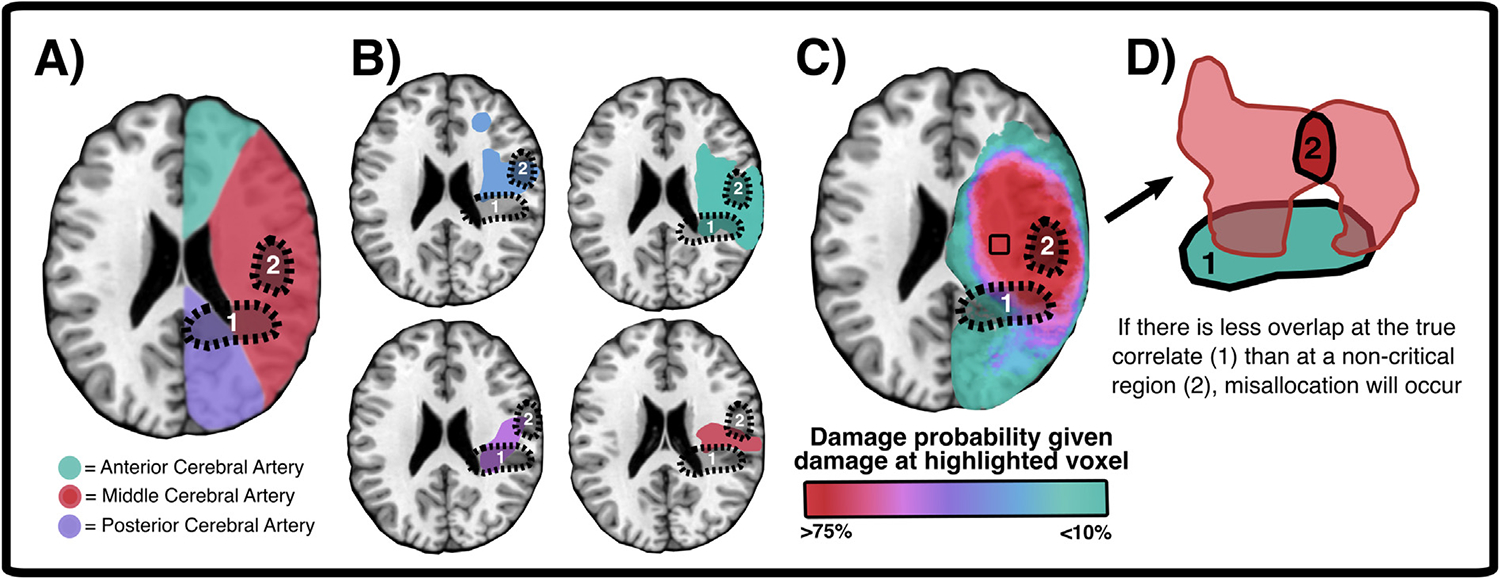
Non-random spatial patterns in lesion location can result in spurious lesion mapping results. (A) Approximate boundaries of major artery vascular territories. (B) Examples of actual lesion boundaries in four individual patients with right middle cerebral artery (MCA) strokes. (C) Group-level overlay (n = 100) with right MCA stroke to illustrate unequal distribution of lesion probability within stroke territories. (D) Example of how unequal overlap in deficit-causing lesions (in red), can cause misallocation. In this case, both lesions overlap with the ‘true’ lesion correlate (1) but have greater spatial homogeneity at a separate, non-critical site (2). In this schematic, area 2 is proximal to the MCA vascular trunk whereas area 1 is a more distal vascular region. This scenario is also present in the real-world lesions presented in (B). When a non-critical area is more likely to be damaged by deficit-inducing lesions than the true correlate, the critical correlate will be mislocalised to the non-critical area. See Mah et al. (2014) for a detailed discussion of this effect.

Despite the popularity of mass univariate lesion mapping, there are a number of limitations associated with its use (Herbet et al., 2015 (Cortex); Inoue et al., 2014; Mah et al., 2014). First, mass univariate approaches consider each individual voxel as an independent statistical test, ignoring the fact that spatially proximal voxels (or more distant voxels within the same vascular territory) are statistically more likely to be damaged together. This means that mass-univariate approaches may not be able to distinguish between voxels that are critical neural correlates of a measured function and voxels which are non-critical, but which are implicated nonetheless by virtue of being included in association with critical voxels (Mah et al., 2014; see Fig. 2). In studies using lesions due to stroke, this effect ultimately biases univariate lesion mapping results toward brain areas located near vascular trunks (Mah et al., 2014). Importantly, non-random lesion distributions can also bias results in studies of other clinical conditions, including tumour, closed head injury, and surgical resection. For example, primary brain tumours are more likely to occur in fronto-temporal cortical and subcortical areas than in other brain regions (Kleihues, 2000; van Kessel et al., 2019). Likewise, in cases of traumatic brain injury the probability of damage is not uniform across all brain areas, though the particular distribution (and direction of bias) may vary across samples (Cristofori & Levin, 2015).

Second, many cognitive functions are not subserved by a single, spatially circumscribed area of brain tissue. Mass-univariate lesion mapping effectively identifies regions that are consistently spared in those without an impairment but damaged in those who are impaired. Therefore, if a function is subserved by multiple regions, each critical module acts as a counter-example for detecting all the other critical modules, leading to very low statistical power. While this limitation obviously does not apply to statistically significant effects, it requires large sample sizes and makes null-effects difficult to interpret. Similarly, many cognitive deficits represent disconnection syndromes caused by severed communications between distant regions rather than by damage to any single area (Thiebaut de Schotten et al., 2008 (Cortex)). The correlates of disconnection syndromes cannot reliably be quantified using mass univariate approaches, because a single critical tract may be disrupted by a wide range of non-overlapping lesions (Rudrauf et al., 2008 (Cortex)).

To address this problem, recent studies have applied tract-level and network-level lesion mapping approaches (Boes et al., 2015; Gleichgerrcht et al., 2017 (Cortex); Rudrauf et al., 2008; Schwen Blackett et al., 2022 (Cortex)). In these methods, disconnection statistics are usually generated by overlaying lesion masks on standard atlases of either white matter tracts (e.g., Johns Hopkins White Matter Atlas) or network-level connections (Griffis et al., 2021). Network-level disconnection severity is most commonly estimated by calculating the proportion of atlas-defined white matter tracts which bilaterally terminate within pairs of atlas-defined grey matter parcels (Griffis et al., 2021). Tract definitions can also be based on in-vivo tractography (Catani & Thiebaut de Schotten, 2008 (Cortex); Forkel & Catani, 2018; Gleichgerrcht et al., 2017), but this approach is less commonly used due to the comparatively limited availability of diffusion imaging (and associated higher cost). For each defined tract or network edge, a score representing the probability of disconnection is calculated. In tract-level analyses, this score represents the degree of disconnection within a single tract. In network-level analyses, scores represent the degree of disconnection within all tracts connecting two given cortical regions (Griffis et al., 2021). Statistical analyses (usually regressions or t-tests) are then conducted to evaluate whether disconnection scores at each tract/edge are associated with the deficit of interest.

Godefroy et al. (2023, Cortex) employed voxel-wise, tract-level, and network-level lesion mapping to explore the correlates of post-stroke executive dysfunction. While voxel-wise analyses revealed executive deficits in association with damage to left hemisphere temporal-parietal areas, tract-level analyses found that impairment was associated with damage to a diverse range of left hemisphere tracts (e.g., anterior commissure, inferior occipitofrontal fasciculus, fronto-striatal tracts) (Godefroy et al., 2023 (Cortex)). These tract-level results were mirrored in network-level analyses, though the latter also suggested that disconnection of the left superior, middle, inferior, and rectus frontal gyri, as well as the thalamus, also contributed significantly to executive performance (Godefroy et al., 2023 (Cortex)). These results highlighted the utility of both tract-level and network-level lesion mapping, as these approaches can identify critical patterns of disconnection which can be missed in voxel-wise analyses.

Although tract- and network-level approaches may provide more reliable insights into the correlates of disconnection syndromes, they are still affected by some of the same limitations outlined above for mass-univariate voxel-wise approaches. Specifically, most disconnection-level analyses are unable to distinguish between contributions of critical connections and those non-critical connections that are damaged alongside the critical tracts. Multivariate lesion mapping approaches have been developed to help mitigate this issue. In multivariate lesion mapping, the synergistic contributions of multiple brain regions are used to generate a single, robust prediction of impairment (Smith et al., 2013). Recent multivariate approaches have relied on a diverse range of algorithmic approaches, including support vector regression and sparse canonical correlation (Malherbe et al., 2018; Pustina et al., 2018; Sperber et al., 2019, 2023).

Multivariate lesion mapping approaches are particularly well-suited to identifying and disentangling multifocal brain regions responsible for regulating specific functions (Pustina et al., 2018; Sperber et al., 2019). For example, previous univariate lesion mapping analyses of unilateral neglect have identified a diverse range of cortical areas and white matter tracts (Moore et al., 2021; Moore, Hearne, et al., 2023; Moore, Jenkinson, et al., 2023). Past work has concluded that this diversity of lesions is likely due to neglect being a multifocal or disconnection syndrome, meaning that multivariate analyses are needed (Herbet et al., 2015; Thiebaut de Schotten et al., 2008). Wiesen et al. (2020, Cortex) addressed this issue by applying multivariate lesion mapping (support vector regression) to identify both voxels and tracts associated with neglect. They found that spatial bias on a line bisection task was primarily associated with damage to the right inferior parietal lobe and basal ganglia. Tract-level analyses revealed that the magnitude of the bias was also predicted by damage to the superior longitudinal fasciculus, arcuate fasciculus, and internal capsule (Wiesen et al., 2020 (Cortex)). Similarly, Ghaleh et al. (2020, Cortex) employed support vector regression multivariate lesion mapping to quantify brain regions associated with verbal working memory impairment. They found that the maintenance of verbal information was supported by distinct cortical regions, depending on the level of task demands (Ghaleh et al., 2020 (Cortex)). In addition, multivariate approaches can disentangle different forms of impairment. For example, Yourganov et al. (2015) demonstrated that the pattern of stroke injury can predict different types of aphasia (Broca’s, Wernicke’s, global, conduction, and anomic), classified based on scores derived from the Western Aphasia Battery. Taken together, these results illustrate the utility of multivariate lesion mapping analyses for disentangling complex, multifocal, and spatially distributed braine–behaviour relationships.

In summary, lesion mapping methodologies have evolved considerably over the past several decades. These methods have been applied to address a wide range of research questions and have contributed important new insights into the functional organisation of the human brain. As we have noted, however, the reliability and reproducibility of lesion mapping results ultimately depend on a wide range of methodological choices outside of the statistical lesion mapping approach used. In the following section, we identify the situations in which lesion mapping analyses can support theoretically meaningful conclusions and highlight some analysis choices we believe can maximise the likelihood of generalisable findings.

## 2. What lesion mapping can (and cannot) tell us about how the brain works

**Fig. 3 –:**
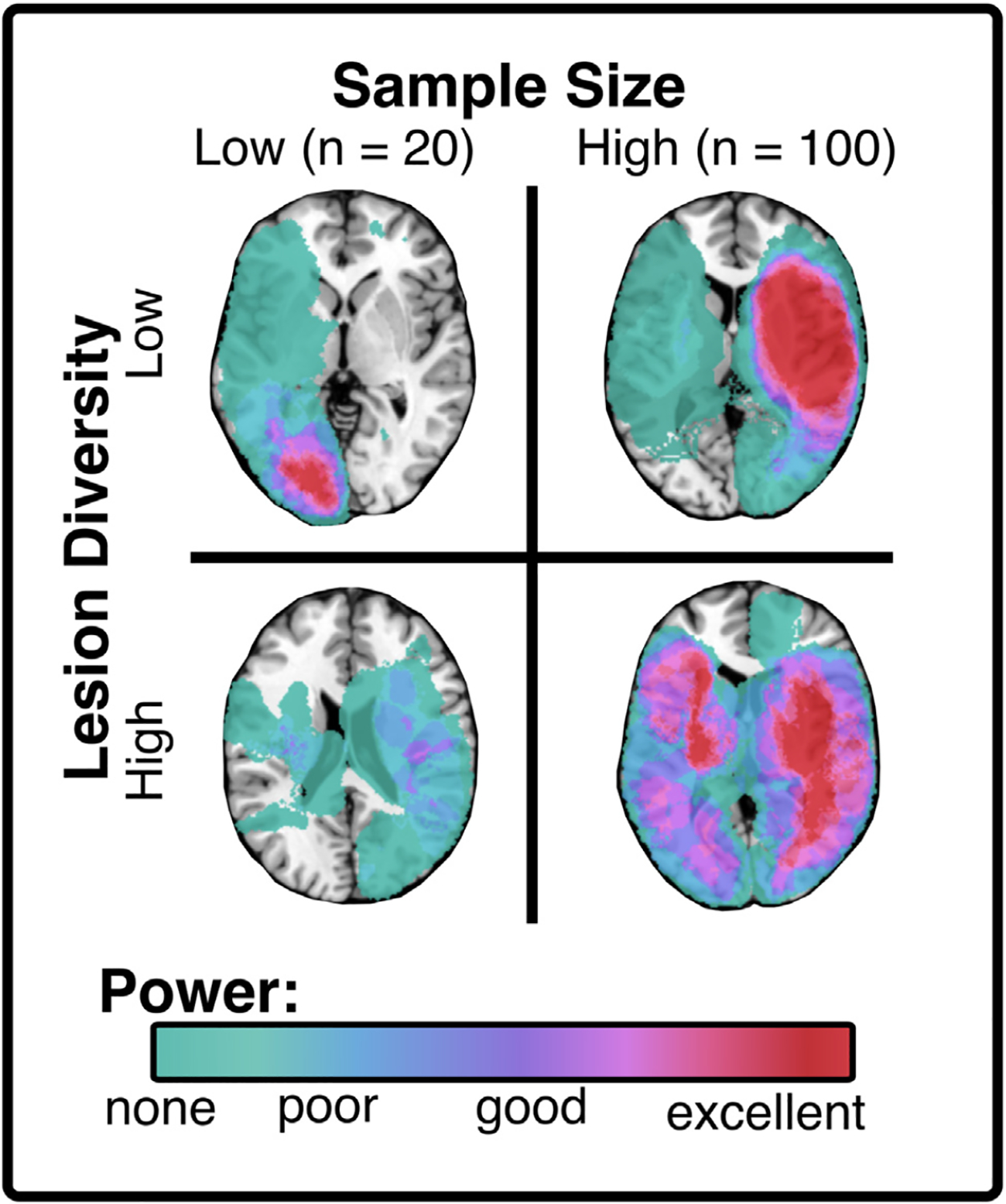
Sample size and diversity of lesion location interact to modulate voxel-wise statistical power in lesion mapping. Each panel shows real lesions from acute stroke survivors to illustrate the effect of sample size and lesion diversity on outcomes. Low diversity samples can yield high statistical power, but only within very restricted areas. Low-diversity lesion samples arise when non-representative samples are recruited by restricting inclusion to specific stroke territories (i.e., right MCA stroke only). Statistical power is optimised in large samples with diverse lesions as this yields the highest average power over the widest range of areas. Importantly, this visualisation does not distinguish between control (behaviourally spared) and test (behaviourally impaired) lesions. In real-world scenarios, power is only optimised when an area is impacted by the lesions of both spared and impaired patients. Note also that a sample size of 100 is comparatively small, and better voxel-wise power will be achieved with much larger samples (e.g., >500).

Any individual methodological approach will be well-suited to address some questions but poorly suited to address others. The power of modern neuroscience is the ability to wield different methods with interlocking strengths and weaknesses to synergistically tackle a research topic. Therefore, a frank recognition of the weaknesses of each method is required. There are several underlying limitations in lesion mapping methodologies that restrict the scope of lesion mapping investigations and thus the insights they can provide into braine–behaviour relationships. First, lesion mapping cannot draw informative conclusions about neural regions which are not affected by enough lesions (Kimberg et al., 2007; Rorden et al., 2009). No inferences can be made about voxels, ROIs, tracts, or networks which are either never damaged or always damaged in the population of interest. Statistical power in lesion mapping is maximised in large and diverse samples (Fig. 3) and when lesion incidence (at a given region) matches the incidence of the deficit of interest (Kimberg et al., 2007). Similarly, lesion mapping investigations are not capable of distinguishing the function of regions that are always damaged together (e.g., within the same lesion). Areas which, in theory, could subserve dissociated functions can never be entirely separated if they are never damaged independently.

Lesion mapping will also have low power to detect deficits caused by the conjunction of damage in multiple separate regions. For example, if a deficit were to result from damage to both areas A and B, individual patients with damage to just one of these areas would effectively provide a counterexample against the necessity of the other region, thereby precluding detection with lesion mapping. In studies employing stroke lesions, archetypal patterns of brain injury and their associated deficits are ultimately driven by the distribution of vascular territories, meaning that functional boundaries can potentially be masked by vascular constraints (Mah et al., 2014; Rorden et al., 2009). Regardless of aetiology, lesion volume will always modulate the probability of impairment. Larger lesions are more likely to overlap with “critical areas”, regardless of whether critical areas involve a single voxel, cortical ROI, or network-level connection (DeMarco & Turkeltaub, 2018). Individual differences in brain morphology, functional organisation and network connectivity can also introduce potentially confounding variability to lesion mapping results. Though some investigations have explored the benefit of incorporating individualized connectivity and functional anatomy data to improve lesion mapping precision (Urbanski et al., 2008, 2011), it is unclear what impact this underlying functional variance has on standard group-level lesion mapping analyses.

Additionally, not all deficits can be expected to map on to a localised pattern of brain damage. For example, depression, anxiety, and fatigue are common following focal brain injury (e.g., stroke, traumatic brain injury), but these deficits may not be causally related to specific lesion locations (Barker-Collo, 2007; Herbet et al., 2015; Lerdal et al., 2009; Litofsky & Resnick, 2009; Schöttke & Giabbiconi, 2015). The probability of developing a clinical mood disorder is modulated by a combination of risk factors (e.g., genetics, socioeconomic factors) and the occurrence of precipitating life events (e.g., stress) (Andersen et al., 1995; Nickel & Thomalla, 2017). In line with this, it is unlikely that the occurrence of post-brain injury mood disorders can be linked to a critical lesion site; instead, they are more likely to be predicted by external risk factors which arise following a brain injury event (Andersen et al., 1995; Nickel & Thomalla, 2017; Williams & Demeyere, 2021). Studies that apply lesion mapping techniques to explore similar deficits (e.g., fatigue, anxiety) may identify apparently significant correlates, but these are more likely to represent artefacts of lesion distributions than theoretically meaningful braine–behaviour relationships. This possibility is consistent with the lack of consensus amongst lesion mapping studies that have sought to identify the neural correlates of depression following brain injury (Jolly et al., 2023; Nickel & Thomalla, 2017).

Lesion-mapping analyses aim to identify neural regions that, when damaged, cause a specific functional deficit (e.g., worse performance on a relevant test of interest). Notably, this approach cannot be meaningfully reversed. In other words, lesion mapping cannot effectively identify areas that, when damaged, are causally related to comparatively better performance (de Haan & Karnath, 2018). Consider, for example, a hypothetical lesion mapping study where the aim is to identify key areas of damage leading to language deficits. Such a study would have a high probability of assigning negative weights (indicating that damage yields better performance) to right hemisphere areas. This does not mean that right hemisphere lesions improve language abilities, but instead suggests that right hemisphere lesions are unlikely to overlap with critical left-hemisphere language networks. This is because the presence of a lesion is a requirement for inclusion, and therefore one can see reliable patterns of injuries that involve vasculature not involved with the task. In this example, the identified negative weights are due to a hemispheric asymmetry of function, but analogous issues can occur at a more local scale. Consequently, specific damage patterns should never be interpreted as being causally related to improved performance, even if they might correlate with improved outcomes. This issue is particularly relevant to studies which aim is to identify neural correlates that predict positive patient cognitive recovery outcomes (Dressing et al., 2020; Forkel & Catani, 2018). A related novel use of lesion mapping is to guide the location of brain injury, for example determining the surgical resection targets than lead to the fewest side effects (Bonilha et al., 2007).

Taken together, there are a wide range of research questions and specific scenarios in which lesion-mapping approaches cannot reasonably be expected to yield theoretically meaningful results. These limitations cannot always be entirely avoided (e.g., volume-related confounds), but it is important to take precautions to mitigate their influence. Researchers wishing to conduct high-quality lesion mapping analyses should be aware of the limitations noted above and critically evaluate from the outset whether the approach is suited to their research endeavour.

## 3. Lesion mapping analysis quality is strongly modulated by methodological choices

Ensuring lesion mapping is well-suited to a specific research question is a key first step in analysis design, but it is not the only choice which ultimately determines study quality. Past research has identified a range of analysis parameters that have the potential to either confound interpretation or maximise the probability of meaningful results. Several past studies have highlighted how specific analysis choices can influence outcomes (de Haan & Karnath, 2018; Ivanova et al., 2021; Mirman et al., 2018; Moore et al., 2023). It is widely recommended that lesion mapping analyses control for lesion volume (DeMarco & Turkeltaub, 2018; Moore et al., 2023). Lesion mapping studies should also aim to employ neuroimaging and behavioural data collected at the same time-point relative to infarct, because differences in cortical reorganisation and behavioural recovery can confound interpretation of results (de Haan & Karnath, 2018). Lesion-mapping studies should also aim to maximise the number and spatial variability of included lesions since accuracy improves with increases in sample size (Ivanova et al., 2021) and lesion mapping can only draw conclusions about regions that are damaged in many patients (Fig. 3). Past work has demonstrated that researchers can leverage routinely available clinical imaging (both CT and MR) to achieve large and representative study samples (Moore et al., 2023; Moore & Demeyere, 2022). Appropriate corrections for multiple comparisons should also be applied in lesion mapping analyses (Mirman et al., 2018). The considerations outlined here are just a few examples of the many possible analysis choices that have the potential to alter mapping outcomes (Moore et al., 2023).

Individual lesion-mapping analyses vary dramatically in quality, but even the highest-quality results can be misinterpreted. Such misinterpretations often take the form of assuming the results of individual analyses are stronger than what they can possibly be. Lesion data can facilitate causal inferences in some, but not all, cases. For example, brain activity measured using electroencephalography (EEG) may be disrupted following local damage, but this disruption might merely be correlated with the presence of a lesion rather than being causally related to a specific cognitive deficit. Even under best-case analysis scenarios, lesion mapping results cannot always be assumed to indicate ground-truth results. There is no gold-standard approach for guaranteeing the accuracy of lesion mapping results. Post-mortem anatomical reconstruction could be applied to precisely quantify lesion boundaries, but such approaches can only provide very broad insights into brain function at a group level. Causal relationships can be tested using methods such as transcranial magnetic stimulation (TMS) and transcranial electrical stimulation (tES), but these approaches are not able to determine the precise boundaries of critical neural regions, particularly when the key sites are located subcortically (Bikson et al., 2012; Kobayashi & Pascual-Leone, 2003; Lefaucheur, 2019). Even when lesion boundaries are precisely defined, depending on aetiology and time since event, functional reorganisation may change normal patterns of brain-behaviour association (de Haan & Karnath, 2018; van Grinsven et al., 2023). This issue is particularly pertinent for patients in whom lesions have developed slowly over time, such as those with brain tumours (Desmurget et al., 2007).

**Fig. 4 –:**
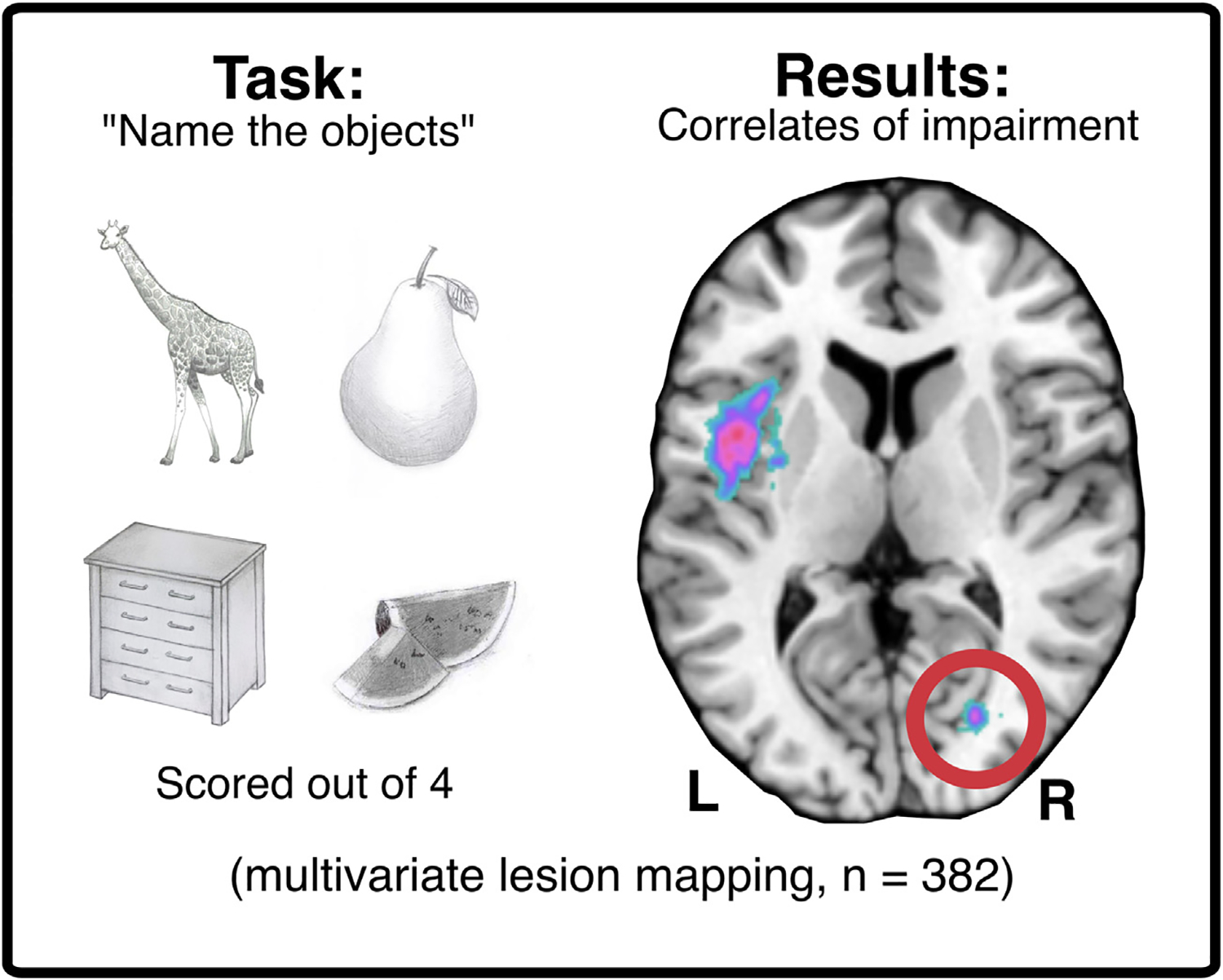
A real-world example of multivariate lesion mapping yielding significant correlates which are not directly related to the function of interest (language). Acute stroke survivors (n = 382) completed the Oxford Cognitive Screen picture naming task (Demeyere et al., 2015). Sparse canonical correlations (multivariate lesion mapping, Pustina et al., 2018) were then applied to identify neural correlates of poor performance. The significant cluster in the left fronto-temporal region aligns with areas traditionally associated with language function, whereas the highlighted cluster in the posterior right hemisphere is associated with visual function (Moore & Demeyere, 2022). This example illustrates how the outcomes that yield the best-fitting models in multivariate lesion mapping may not always provide helpful insight into function. The “false positive” correlates in this example are easily identifiable via visual inspection, but this may not always be the case for more complex or poorly understood functions.

Multivariate lesion-mapping approaches may mitigate some of the spatial biases present in univariate analyses, but they are still susceptible to some degree of mislocalisation (Ivanova et al., 2021; Mirman et al., 2018; Sperber et al., 2019). While machine-learning based lesion-mapping approaches may provide accurate predictions for deficits based on lesions, the results they produce do not necessarily provide insight into the disorder of interest. Mainly, multivariate, machine-learning based lesion mapping approaches can, in theory, use factors unrelated to the function of interest to distinguish between spared and impaired patients. For example, in a hypothetical lesion-mapping analysis of aphasia, a multivariate approach might reveal significant voxels in the primary visual cortex. Although few would argue that early visual areas are necessary for language, visual field deficits might well be associated with worse performance on language tests that require intact vision (e.g., picture naming, reading) (Demeyere et al., 2016; Demeyere et al., 2015) (Fig. 4). Multivariate approaches capitalise on these relationships to improve model fits in a manner that is not always transparent to researchers. There is inherent tension between the accuracy of generated behavioural predictions and the strength of anatomical inferences which can be drawn. In other words, more detailed predictive models provide more accurate classification results, but as the number of predictors increases, the value contributed by any individual predictor decreases. It is therefore important to interpret multivariate lesion mapping results with caution.

It is critical to assess the reliability and accuracy of results through the degree of consensus present between multiple independent analyses across different patient groups. Lesion-mapping results indicating true correlates can be expected to be consistent across multiple different analysis types and sample groups, while less reliable results will fluctuate across studies (Bates et al., 2003; de Haan & Karnath, 2018; Ivanova et al., 2021). Univariate and multivariate methods provide different types of answers, and therefore should be seen as complementary rather than opposing approaches. Brain injuries can involve large, spatially contiguous regions where injury to one voxel is highly correlated with injury to its neighbour. By design, machine-learning multivariate methods are more robust to these highly correlated predictors than traditional statistical models. Faced with features that provide common information, however, many multivariate models may give particular weight to one feature while ignoring another feature that is predictive but does not add any unique information to the model. For example, if two neighbouring voxels provide the same level of predictive information for a training set (e.g., are both damaged in all impaired patients) some multivariate approaches will arbitrarily select one voxel to use and one voxel to exclude, because the information in the excluded voxel would not significantly add to the information already captured by the model.

Other approaches show the same instability but do this by preferentially weighting one feature over another similar feature. Although some approaches are comparatively less susceptible to this type of problem (e.g., by using algorithms that adjust the weights of similar features in tandem), their interpretability can be compromised by diluting feature importance across many similar features. This can lead to counterintuitive assignments, in which relatively low weights are given to features that are highly predictive on their own, and relatively high weights are given to features with low predictive power. In neuroimaging, additional problems with interpreting feature weights can arise because features tend to grossly outnumber samples. In such settings, it becomes increasingly difficult for multivariate models to distinguish between features, making it more likely they will assign weights that are more sensitive to random variations in the training data and noise. In contrast, univariate methods test each voxel in isolation. It might therefore be worth thinking of univariate statistics as creating spatial maps of candidate brain areas which are most related to a certain behaviour, while multivariate approaches use all brain areas to provide the most accurate prediction of impairment and outcome. For this reason, the addition of features that correlate with impairments – such as lesion volume, age at injury, and residual white matter integrity – often reduces the statistical power of univariate methods yet improves the predictive capabilities of multivariate methods.

## 4. Some, but not all, lesion mapping applications have translational value

In recent years, many studies have harnessed lesion-mapping techniques to generate findings with potential practical value to clinicians. These lesion-mapping studies have aimed to improve diagnosis of cognitive impairments, improve prognostic models, and identify patients who are unlikely to recover without targeted interventions. Each of these research avenues has potential value for improving clinical care, but only if lesion-mapping analyses are correctly applied and interpreted.

Several previous investigations have used lesion-mapping results to develop visual lesion-location rating scales to help clinicians identify patients who may have a cognitive impairment after an acquired brain injury. This approach has been used most commonly to predict deficits following stroke-induced lesions (e.g., Weaver et al., 2021) but some studies have explored similar approaches in patients with traumatic brain injury and brain tumours (Cristofori & Levin, 2015; van Grinsven et al., 2023; Wick et al., 2008). There are several issues with this application of lesion-mapping findings for diagnosing concurrent cognitive impairment. The most obvious issue is that the outcome being predicted (concurrent cognitive impairment) is most easily measured directly. The established gold-standard method for characterising cognitive impairment is to use validated neuropsychological testing (Quinn et al., 2021). Next, in real-world patient samples, many impaired patients may have lesions that do not overlap with the significant voxels identified by lesion mapping analyses (and vice versa) (Moore & Demeyere, 2022). Third, within stroke samples, acute neuroimaging is typically conducted using CT scanning, given its wide availability, tolerability, low cost, and the primary aim of detecting haemorrhagic stroke on admission to determine treatment. However, acute CT brain imaging has a very high false negative rate in ischaemic stroke, meaning that lesion boundaries are not always clearly visible to clinicians (Urbach et al., 2000). From a pragmatic viewpoint, even if high-fidelity imaging is available, the time and expertise required for the creation of lesion maps, through manual delineation or (semi-)automated methods, is not likely to be available to clinicians. Past work has also suggested that clinician knowledge of lesion location may reduce the sensitivity of observational cognitive impairment diagnosis in cases where impaired patients exhibit less prototypical lesion locations (Moore et al., 2019). In sum, given the uncertainty in the reliability of lesion mapping results, the variability present in clinical imaging, and the data which could be practically accessed by clinicians in real-world environments, lesion mapping based diagnoses cannot be considered a feasible alternative to neuropsychological cognitive assessment.

Similarly, many previous studies have employed lesion mapping approaches to search for lesion-location predictors of patient recovery outcomes. This line of work is very promising, as several past studies have identified critical regions that, when damaged, are associated with a disproportionately reduced degree of functional recovery over time (Bowren et al., 2022; Dressing et al., 2021; Forkel & Catani, 2018). This work has great translational potential as it can facilitate early identification of patients who may require more intensive rehabilitation (though we need to consider carefully the risk of restricting therapy for those with more positive predicted outcomes). Additionally, it remains important for studies aiming to identify correlates of recovery outcomes to consider that lesion location is not the only factor that predicts recovery trajectories. A more comprehensive approach, taking into account the full gamut of predictors and balancing predictions from an ideal model against more pragmatic prediction modelling approaches, is ultimately what is needed.

Brain health (outside of lesion location) is a key factor which cannot be ignored when predicting cognitive outcomes (Hobden et al., 2023; Hobden et al., 2022). Lesion volume, lesion multiplicity and reoccurrence all matter in addition to lesion location and more broadly, age, education, social economic status, premorbid decline, and life-long exposure to vascular risk factors all impact risk predictions for long term cognitive outcomes (Rost et al., 2022). Each of these established predictive data sources is more practically accessible to clinicians than lesion-location based predictors (Milosevich et al., 2023). Detailed lesion-location metrics (e.g., tract disconnection, voxels impacted) are computationally intensive to generate as they require manual lesion delineation, spatial normalisation, and detailed normative atlas comparisons by a trained expert before they can be effectively interpreted (de Haan & Karnath, 2018; Griffis et al., 2021; Moore, 2022). These requirements do not diminish the utility of lesion-location based findings, but rather necessitate that any lesion-location based predictor must add predictive information significantly over and above that which is already provided by more practical data sources in order to be clinically useful and justify the additional costs for the additional explanatory contribution in a health economics evaluation.

With advances in treatments for brain injury, tumour, neurosurgery, and stroke, there are more people living with brain injury. This means that the quantity and quality of lesion-displaying neuroimaging is growing dramatically, providing the resources necessary to facilitate a wide range of clinically relevant lesion mapping investigations. Cognitive impairment is a key predictor of poor recovery outcome (Bisogno et al., 2023 (Cortex); Milosevich et al., 2023; Nys et al., 2006), and understanding the neurological causes of these deficits is an important precursor to designing targeted rehabilitation strategies. Lesion mapping analyses, when conducted effectively, are a promising avenue for helping to inform targeted rehabilitation pipelines and improve prognostic modelling. This, in turn, may help reduce inefficient health resource usage and improve the quality of information clinicians are able to provide to patients (Stinear et al., 2017).

## 5. Practical guidance for future high-quality lesion mapping studies

**Fig. 5 –:**
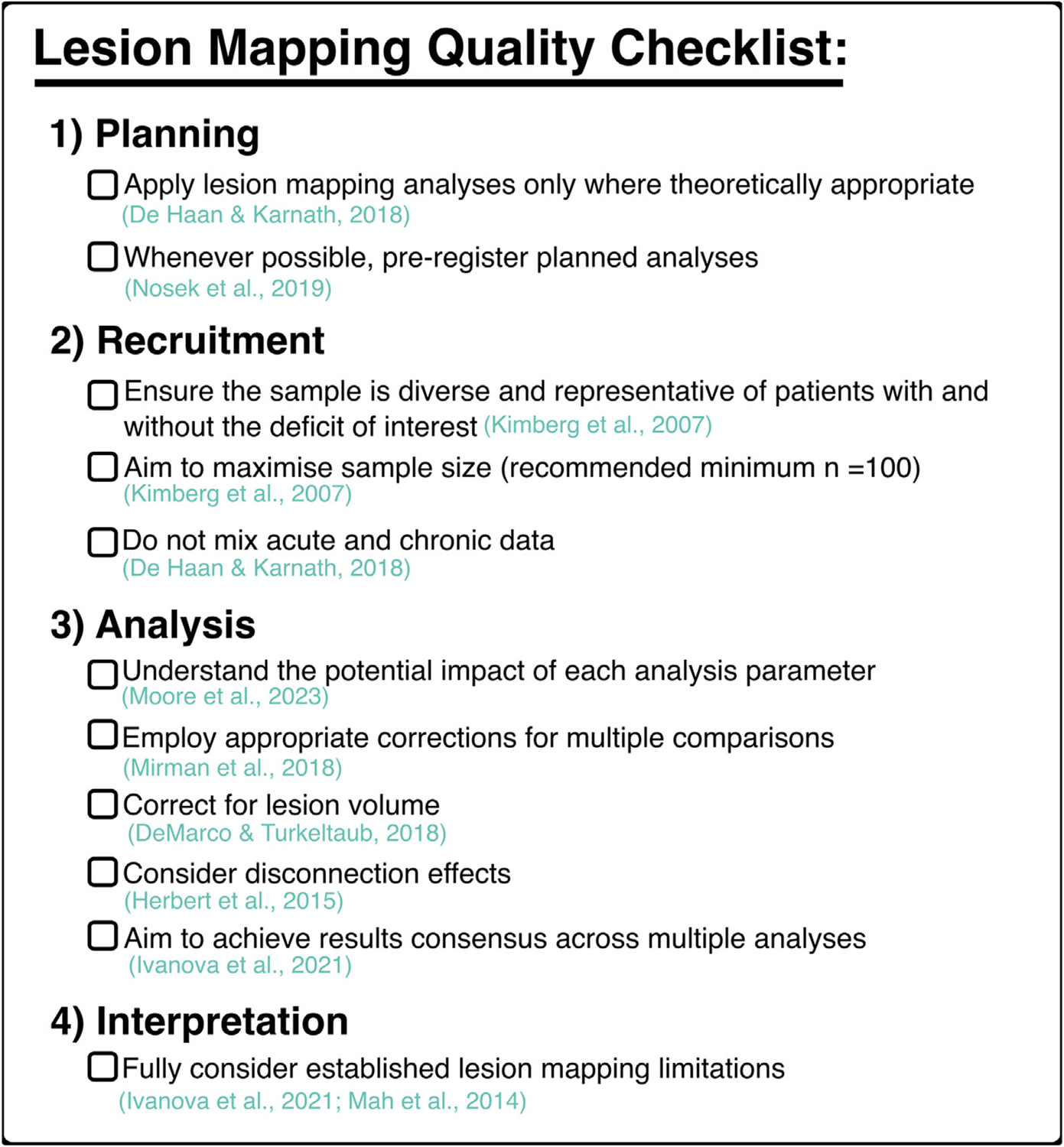
A checklist outlining established criteria for conducting high-quality lesion mapping investigations. Each criterion is accompanied by a key reference which explains the importance of each standard in detail. Importantly, the exact method employed for each analysis will vary based on the relevant research question. However, these general principles remain applicable to all future studies.

**Fig. 6 –:**
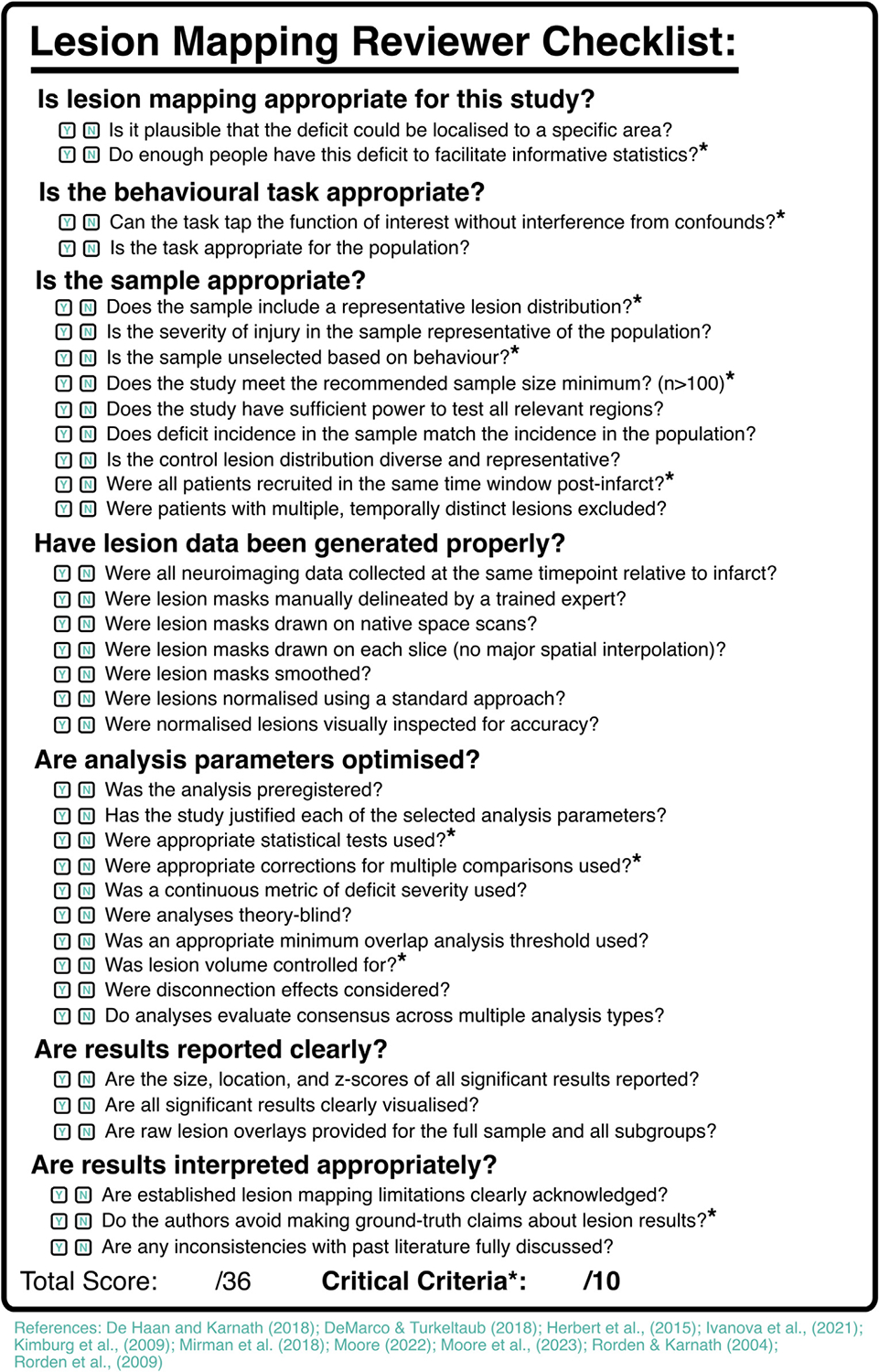
Checklist for reviewers aiming to assess the quality of lesion mapping analyses. Critical criteria, when unmet, indicate a high risk of biased or confounded lesion mapping results. Other criteria, when met, improve the quality of lesion mapping analyses but do not risk significant confounds when not met. A detailed user guide for this checklist (and scored real-world examples) are available at https://osf.io/4pk8a/.

Lesion mapping remains a valuable tool but, as we have explained, it is more likely to yield theoretically and practically useful results if it is applied correctly. To this end, we have compiled a quality-control checklist for best-practice lesion mapping (Fig. 5), the goal of which is to facilitate high-quality lesion mapping investigations. Exact analysis design will vary based on the specifics of individual research questions, but the general criteria outlined in the checklist should be met by all lesion mapping studies. If these established criteria are not met lesion mapping analyses should not be conducted or should be explicitly treated as speculative and unlikely to produce generalisable results. Pre-registration is an important tool for researchers aiming to design high-quality analyses. It allows researchers to plan and justify each chosen analysis parameter in advance of data collection, in order to ensure their choices are made based on best-practice recommendations rather than on which choices result in significant findings. We have also constructed a detailed checklist for reviewers aiming to assess the quality of lesion mapping investigations contained in research manuscripts or grant applications (Fig. 6). This checklist identifies 10 criteria which must be met for lesion mapping results to support meaningful theoretical inferences. The checklist also defines 26 criteria which have been demonstrated to improve the quality of lesion mapping analyses.

## 6. The next steps for lesion mapping

Lesion mapping methodologies have evolved considerably over the past few decades, but this evolution is far from complete. There are several key unaddressed research questions which have the potential to facilitate more informative interpretation of lesion mapping results and to continue advancing lesion mapping analysis methods. First, future research should aim to quantify the accuracy of lesion mapping methods and identify factors that affect accuracy. Simulation-based lesion mapping studies offer a promising approach for accomplishing this goal (Ivanova et al., 2021; Mah et al., 2014). In simulation studies, real lesion masks are used to generate simulated behavioural scores relative to a defined anatomical target (e.g., a voxel or ROI). When lesion mapping is run using these real lesions and simulated scores, the accuracy of each analysis relative to the underlying target can be precisely quantified (Mah et al., 2014). Past studies have used this approach to compare the accuracy of different statistical approaches (e.g., univariate vs multivariate), different statistical corrections, and other analysis parameters (Ivanova et al., 2021; Mirman et al., 2018; Moore et al., 2023). Critically, this method has the potential to quantify how individual analysis choices, lesion distributions, and underlying neural correlates interact to modulate analytic outcomes. This, in turn, will provide a solid foundation for understanding the reliability of results derived from actual lesion mapping analyses, and will help guide future studies toward higher-quality lesion mapping analyses.

Future work should also aim to develop methods for understanding which pattern of underlying neural correlates would produce the significant results generated by lesion mapping analyses. In other words, lesion mapping could be improved by developing methods for better understanding which underlying correlates might cause the observed results, rather than interpreting the observed results themselves. This goal could be accomplished by leveraging simulation-based approaches to determine what results clusters would look like in a specific lesion distribution, if a range of potential regions were responsible for producing critical correlates. This approach is computationally intensive, but has potential to remedy the issue of results misallocation, which is a fundamental limitation across all currently used lesion mapping analyses.

There is a clear need for further large-scale, multi-method studies to provide convergent evidence on braine–behaviour relationships. For example, lesion mapping results can be directly compared with data from functional neuroimaging to provide a more complete view of how lesions affect the spatial and temporal dynamics of the brain, and how these changes relate to specific cognitive impairments (Fox et al., 2014; Urbanski et al., 2008). Future research should also explore new avenues for considering both voxel-level and network-level contributions in the same analysis. In current methods, voxel-level and disconnection-level lesion factors are almost exclusively considered in isolation, despite clear interactions between these anatomical levels. It is theoretically plausible that disconnection patterns could be leveraged to generate more precise predictions for voxel-wise critical correlates, but methods for accomplishing this have not yet been established.

Furthermore, integrating lesion-based information in advanced statistical predictions for individual risk and trajectory predictions alongside a wide array of relevant predictors would revolutionise its clinical translational potential. Specifically, future studies should aim to quantify the prognostic value of both broad lesion location (e.g., stroke territory) as well as detailed lesion anatomy (e.g., overlap with critical voxels, tracts, or networks) for predicting long-term patient outcomes. More pragmatically-focused work is also needed to establish avenues for making informative lesion metrics more practically available to clinicians. For example, there is a clear need for automated tools capable of deriving lesion and brain health metrics from clinical scans. This work is necessary before any lesion-based prognostic value can be used in a real-world context to improve patient prognostic predictions.

Overall, lesion mapping is an exceptionally valuable tool for understanding braine–behaviour relationships. While these methods offer important insights into the necessary neural correlates supporting behaviour and cognition, recent research has highlighted that even the most computationally advanced lesion mapping methods are not always accurate. These findings do not undermine the value of lesion mapping methods, but instead highlight important avenues for future research where we should aim to further improve lesion mapping methods by designing new approaches for addressing and overcoming the sources of bias highlighted in past research. We need to ensure that lesion mapping studies meet established criteria, be more cautious about when we use lesion mapping approaches and how we interpret their outcomes and develop a deeper understanding of how and why different methodological choices affect outcomes.

## Data Availability

There are no data associated with this manuscript.
